# Paramedic Acute Stroke Treatment Assessment (PASTA): study protocol for a randomised controlled trial

**DOI:** 10.1186/s13063-018-3144-z

**Published:** 2019-02-12

**Authors:** Christopher I. Price, Lisa Shaw, Peter Dodd, Catherine Exley, Darren Flynn, Richard Francis, Saiful Islam, Mehdi Javanbakht, Rachel Lakey, Joanne Lally, Graham McClelland, Peter McMeekin, Helen Rodgers, Helen Snooks, Louise Sutcliffe, Pippa Tyrell, Luke Vale, Alan Watkins, Gary A. Ford

**Affiliations:** 1 Stroke Research Group, Institute of Neuroscience, Newcastle University, 3-4 Claremont Terrace, Newcastle upon Tyne, NE2 4AE UK; 2 Lay investigator. Contact via: Stroke Research Group, Institute of Neuroscience, Newcastle University, 3-4 Claremont Terrace, Newcastle upon Tyne, NE2 4AE UK; 3 Institute of Health and Society, Newcastle University, Baddiley-Clark Building, Richardson Road, Newcastle upon Tyne, NE2 4AX UK; 4 College of Medicine, Swansea University, Singleton Park, Swansea, SA2 8PP Wales; 5 Newcastle Clinical Trials Unit, Newcastle University, 1-4 Claremont Terrace, Newcastle upon Tyne, NE2 4AE UK; 6 North East Ambulance Service, Bernicia House, Goldcrest Way, Newburn Riverside, Newcastle upon Tyne, NE15 8NY UK; 7 Faculty of Health & Life Sciences, Northumbria University, 2nd floor Northumberland Building, Newcastle upon Tyne, NE1 8ST UK; 8 Newcastle upon Tyne Hospitals NHS Foundation Trust, Royal Victoria Hospital, Queen Victoria Road, Newcastle upon Tyne, NE1 4LP UK; 9 Stroke Medicine, Clinical Sciences Building, Salford Royal Hospitals’ NHS Foundation Trust, Salford, M6 8HD UK; 10 Medical Sciences Division, University of Oxford, and Oxford University Hospitals NHS Foundation Trust, Level 3, John Radcliffe Hospital, Oxford, OX3 9DU UK

**Keywords:** Stroke, Pre-hospital research, Paramedics, RCT, Economic evaluation, Parallel process evaluation

## Abstract

**Background:**

Despite evidence from clinical trials that intravenous (IV) thrombolysis is a cost-effective treatment for selected acute ischaemic stroke patients, there remain large variations in the rate of IV thrombolysis delivery between stroke services. This study is evaluating whether an enhanced care pathway delivered by paramedics (the Paramedic Acute Stroke Treatment Assessment (PASTA)) could increase the number of patients who receive IV thrombolysis treatment.

**Methods:**

*Study design:* Cluster randomised trial with economic analysis and parallel process evaluation.

*Setting:* National Health Service ambulance services, emergency departments and hyper-acute stroke units within three geographical regions of England and Wales.

*Randomisation:* Ambulance stations within each region are the units of randomisation. According to station allocation, paramedics based at a station deliver the PASTA pathway (intervention) or continue with standard stroke care (control).

*Study intervention:* The PASTA pathway includes structured pre-hospital information collection, prompted pre-notification, structured handover of information in hospital and assistance with simple tasks during the initial hospital assessment. Study-trained intervention group paramedics deliver this pathway to adults within 4 h of suspected stroke onset.

*Study control:* Standard stroke care according to national and local guidelines for the pre-hospital and hospital assessment of suspected stroke.

*Participants:* Participants enrolled in the study are adults with confirmed stroke who were assessed by a study paramedic within 4 h of symptom onset.

*Primary outcome:* Proportion of participants receiving IV thrombolysis.

*Sample size:* 1297 participants provide 90% power to detect a 10% difference in the proportion of patients receiving IV thrombolysis.

**Discussion:**

The results from this trial will determine whether an enhanced care pathway delivered by paramedics can increase thrombolysis delivery rates.

**Trial registration:**

ISRCTN registry, ISRCTN12418919. Registered on 5 November 2015.

**Electronic supplementary material:**

The online version of this article (10.1186/s13063-018-3144-z) contains supplementary material, which is available to authorized users.

## Background

Stroke is responsible for a high global burden of mortality and disability [1]. In the UK it remains the third leading cause of death and the single largest cause of adult disability with an economic impact of approximately £7 billion per year [2].

The most widely used cost-effective emergency treatment is intravenous (IV) thrombolysis using recombinant tissue plasminogen activator for selected ischaemic stroke cases within 4.5 h of symptom onset [3]. Despite evidence from clinical trials, related guidelines and policy, national audit continues to show large variations in the rate of IV thrombolysis delivery between services and diurnal variations within services [4, 5]. Outcomes are highly time dependent [3, 6]. Within 60–90 min of symptom onset, only four suitable patients need to be treated for one person to be free from disability, whereas at 270 min the same impact would require nine patients to be treated [3]. In 2014, only 11% of total stroke admissions in the National Health Service (NHS) were treated with IV thrombolysis against an aspirational target of 20%, with a median door to treatment time of 54 min despite a target of < 40 min [4]. Benefits for patients and social care resources would be substantially improved if more eligible patients recieved IV thrombolysis, and if they were treated sooner.

Most stroke services have found it challenging to improve IV thrombolysis rates and reduce treatment delays in hospital (i.e. door to needle time (DTNT)), particularly because of access to brain imaging (i.e. door to scan time (DTST)). Brain imaging is a vital component of assessment to exclude haemorrhagic stroke and patients with established ischaemic changes where IV thrombolysis would be futile and potentially harmful. International clinical guidelines state that brain imaging should be performed immediately when a patient with IV thrombolysis potential arrives [7, 8]. An urgent scan is also indicated for other patients presenting with stroke symptoms, including those taking anticoagulation medication and those for whom a haemorrhage is suspected. Urgent treatments for these patients can include reversal of anticoagulation, intravenous medication to lower high blood pressure and possible neurosurgical intervention [8]. Although across the NHS brain imaging was achieved within 1 h for 43% of stroke admissions in 2014, there was wide variation between services, and the low IV thrombolysis rate suggests that patients with the most to gain from rapid radiological assessment were not actively being identified [4]. An improvement in the early identification of patients who meet the criteria for an urgent scan could lead to a cost-effective reduction in dependency, mainly through an increase in the rate and speed of IV thrombolysis treatment, but also by improving access to other treatments and organised stroke care.

To date, most service interventions to increase IV thrombolysis and reduce delays have focussed on responses after patient admission to the Emergency Department (ED) or Hyper-Acute Stroke Unit (HASU). Where improvements have been seen, this typically reflects highly resourced, large volume urban centres, but even these may rely upon initial patient assessment by nursing and junior medical staff at nights and on weekends. In some settings, the standard approach is remote stroke specialist assessment by video link or telephone, increasing the reliance upon non-specialist staff for rapid and accurate information collection and communication at the bedside.

In the pre-hospital setting, there is good evidence that the paramedic sensitivity for stroke identification using the Face Arm Speech Test (FAST) is equivalent to that of non-specialist ED staff [9] and that ambulance contact to the ED to provide advance notice of admission (pre-notification) can have a positive impact [10, 11]. However, despite existing operational guidelines within ambulance services to encourange identification of suspected stroke and symptom onset time, pre-notification does not occur systematically in clinical practice, and the significance of other information collected by paramedics for IV thrombolysis decision making may not be realised during patient handover at hospital (e.g. current medication and recent medical history). Although healthcare policy supports ongoing development of the paramedic role [12], there has been no rigorous examination of how paramedics could best contribute to improving DTNT and DTST. A Swedish randomised trial showed that IV thrombolysis rates and hospital treatment delays significantly improved after paramedics were provided with training and the emergency status of stroke ambulance dispatch was raised [13]. An observational study in Helsinki collecting data before and after a simple training package for ambulance personnel showed that on-scene time reduced by an average of 2.5 min, but there was insufficient power to assess the impact on IV thrombolysis treatment rates [14]. An alternative model of ‘mobile stroke units’ (adapted ambulances with computerised tomography (CT) scanner and neurologist on board or video link to a stroke specialist) has been shown to reduce service-level call to needle times (CTNTs) by 15 min in dense urban areas, but the impact on outcomes must be balanced against the additional costs and technical challenges [15–17]. As the ambulance transfer time from scene to hospital in most of England is already short, this model is unlikely to be adopted [18].

Feedback from the hospital team to paramedics about individual stroke assessments appears to improve future adherence to pre-hospital protocols including pre-notification [19], but no process exists to routinely facilitate this. For unselected emergency admissions, a paramedic-initiated standardised communication approach appears to improve the accuracy and efficiency of handover [20], and there may be value in a format which is stroke specific. In acute medical settings there is increasing evidence that checklists are effective for improving patient safety and protocol adherence [21], and a paramedic protocol could include simple questions to prompt important hospital care processes (e.g. confirm that communication has been established with the stroke specialist).

This study will evaluate the clinical and cost effectiveness of a paramedic-initiated ambulance care pathway which seeks to facilitate the hospital assessment of patients presenting with acute stroke symptoms in order to specifically increase IV thrombolysis rates and reduce treatment delays. Patient and professional views about the care pathway will also be described. This Paramedic Acute Stroke Treatment Assessment (PASTA) pathway consists of structured pre-hospital information collection, prompted pre-notification, structured handover of information in hospital, assistance with simple tasks during the first 15 min of hospital assessment, a checklist to confirm progress after 15 min and a paramedic request for feedback before departure. The pathway has been developed through systematic review of the literature regarding enhanced roles of paramedics as well as developmental workshops with clinicians and support personnel in order to define professional roles and operational boundaries which are feasible whilst maximizing value for patient care.

## Methods

### Study aim and objectives

#### Aim

The aim of the study is to determine the clinical and cost effectiveness of an enhanced PASTA pathway.

#### Objectives

The study objectives are as follows:

- To determine whether the PASTA pathway improves patient care and outcomes. Primary outcome: proportion of patients receiving IV thrombolysis. Secondary outcomes: stroke severity 24 h after IV thrombolysis (National Institutes of Health Stroke Scale (NIHSS) [22]), complications after IV thrombolysis, inpatient mortality, discharge destination, discharge dependency (modified Rankin Scale (mRS) score [23]), assistance at discharge, 90 day dependency (mRS), 90 day destination, assistance at 90 days
- To describe the impact of the PASTA pathway on time intervals from emergency call and hospital admission to first brain imaging, IV thrombolysis treatment (if given), HASU admission and formal assessment of swallowing safety
- To describe the number and subsequent diagnoses of suspected stroke patients who travelled to hospital with a study paramedic but following assessment at hospital were not given a diagnosis of stroke (‘stroke mimics’)
- To determine the cost effectiveness of the PASTA pathway relative to standard NHS stroke care
- To report patient and professional views and experiences about the PASTA pathway.

### Study design

**Fig. 1:**
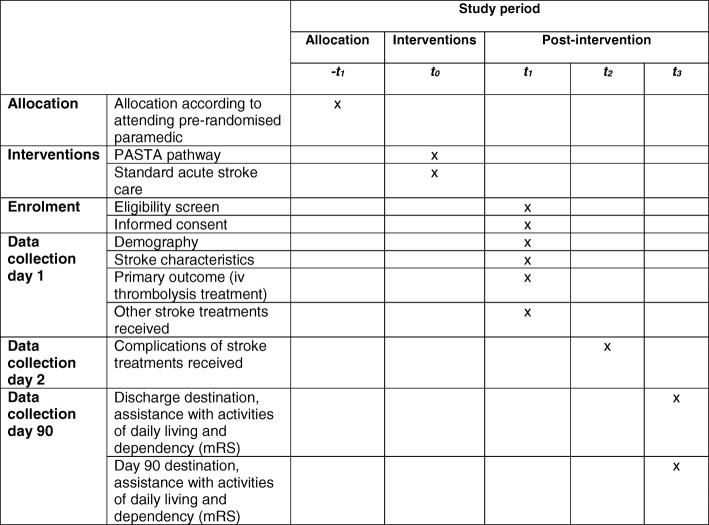
SPIRIT schedule of enrolment, interventions and assessments

This study is a cluster randomised controlled trial (RCT) with embedded economic analysis and process evaluation. Participants receive either the PASTA pathway (intervention group) or standard stroke care (comparison group). The study is presented according to the Standard Protocol Items: Recommendations for Interventional Trials (SPIRIT) guidelines. Figure 1 shows a SPIRIT schedule of enrolment, interventions and assessments. The SPIRIT 221 checklist is provided in Additional file 1.

### Study setting

The study is taking place within ambulance services and a selected number of receiving hospital sites. Hospital sites within each region represent a range of service designs and historical efficiencies in the provision of acute stroke care. All hospital sites receive emergency stroke admissions and provide 24-h access to brain imaging and a stroke specialist opinion in order to make an IV thrombolysis decision.

### Randomisation

PASTA is a cluster RCT in which the unit of randomisation is an ambulance station. Clusters comprise the paramedics based within stations. Prior to the start of the trial within each ambulance service, those ambulance stations which feed into a study hospital were randomised to delivering the PASTA pathway or to continue with standard stroke care.

Stations within each service were stratified according to size (categorised as small, medium or large according to the personnel and resources available) and distance from the nearest study hospital admitting stroke patients (distance categorised as near or far, reflecting the local geography of each ambulance service). The use of these stratifying variables ensured that PASTA care paramedics (intervention) and standard care paramedics (control) were approximately equally matched in terms of operational characteristics.

Paramedics based at stations randomised to continuing standard care were informed that there is an ongoing study of pre-hospital assessment for stroke patients but were not given any further information about the intervention. Paramedics based at stations randomised to deliver the PASTA pathway were asked to complete study-specific training (see also the ‘Staff training and awareness’ section later in the paper). All paramedics could opt out of the study if they wished.

### Study treatments

#### PASTA pathway (intervention group)

The PASTA pathway is delivered by study-trained intervention group paramedics. The pathway is delivered to the following patients: aged 18 years and over; FAST [9] positive or any presentation of new focal neurological symptoms which indicate acute stroke in the paramedic’s routine clinical judgement; within 4 h of last known to be without new stroke symptoms in the paramedic’s judgement; admission to a study hospital.

**Fig. 2:**
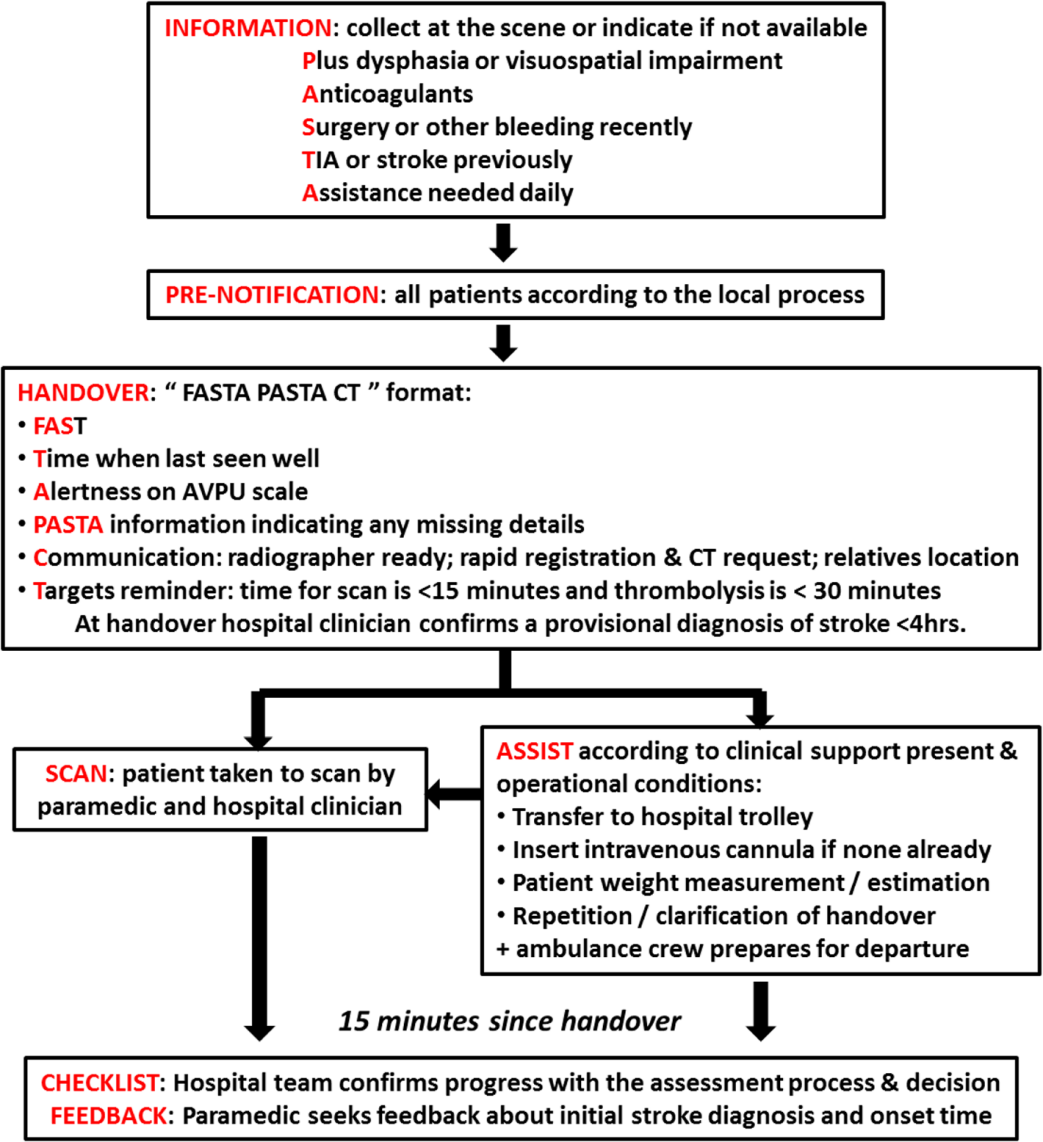
PASTA pathway

A summary of the PASTA pathway is shown in Fig. 2. It consists of the following stages:

1. *Information*. The paramedic will seek additional information at the scene which is routinely considered during IV thrombolysis treatment decisions but is typically not obtained until after hospital admission. This will include:
  The presence of language (dysphasia) or visual (visuospatial) problems during a simple clinical examination, which may indicate a level of stroke severity more likely to be considered for IV thrombolysis treatment than FAST symptoms alone
  Prescription of anticoagulant medication, which would require additional urgent measurement of blood clotting indices before a IV thrombolysis decision could be made. This medication is also an additional indication for urgent brain imaging by itself, as a stroke due to haemorrhage would trigger urgent reversal of its effects
  A recent medical history of surgery or bleeding, which might exclude IV thrombolysis treatment because of an increased risk of uncontrollable haemorrhage
  Any previous medical history of transient ischaemic attack (TIA) or stroke, which could assist interpretation of brain imaging and specialist evaluation of the risk versus benefit of IV thrombolysis treatment
  The current level of dependency according to whether the patient requires direct assistance with feeding or walking, in order to judge the value of administering IV thrombolysis treatment relative to the effects of the new stroke

Within the pathway these information categories will be prompted by the acronym PASTA, which represents Plus dysphasia or visuospatial impairment; Anticoagulant medications; Surgery or other bleeding recently; TIA or stroke previously; Assistance needed daily.

2. *Pre-notification*. During emergency transfer the paramedic will always be expected to attempt a pre-alert to the destination hospital in accordance with existing local arrangements. Although pre-notification is already a component of standard care, compliance is variable. Existing arrangements differ within regional ambulance services and individual hospitals, e.g. direct telephone contact from paramedic, or via ambulance dispatch to ED or to HASU. The individual hospital response can vary according to the timing of the admission (e.g. the patient may be received by a specialist stroke team during weekday office hours and by ED clinicians at other times). The PASTA pathway does not change local processes but routinely prompts standard pre-notification.
3. *Handover*. On arrival at the hospital’s designated location for stroke admissions, the paramedic will provide a standardised handover of stroke-specific information (FAST, onset time, alertness and PASTA details) to the hospital team, indicating any items which were unavailable at the scene. The paramedic will ask the receiving hospital team whether it is possible to go straight to the CT scan if the radiographer is ready and will remind them about ideal time targets for scan (< 15 min) and IV thrombolysis treatment (< 30 min). A member of the ambulance crew will assist the team with rapid registration of the patient’s details on the hospital patient administration system. Prior to CT scan, a hospital clinician will always confirm that the provisional diagnosis is likely to be stroke within the previous 4 h. In addition, the paramedic will inform the team about the known location of any relatives in order to facilitate information gathering, communication and treatment decisions.
4. *Scan*. If the CT scan is immediately available, the paramedic will assist with patient transfer to the scan room accompanied by at least one member of the hospital team. It will be a local decision whether the patient is first transferred onto a hospital trolley, but any delays should be minimised.
5. *Assist*. If the CT scan is not immediately available, the hospital team will continue with urgent care of the patient according to the local service protocol, and the patient will be transferred onto a hospital trolley. If ambulance service operational conditions are suitable and the hospital team require assistance, the paramedic can assist with the following tasks: insertion of an intravenous cannula if not already inserted; determining the patient’s weight from assisting with transfer onto measurement scales or contributing towards a team estimation of weight; and repetition/clarification of clinical information (e.g. repeating the handover to a member of the stroke team). If the scan becomes available within 15 min of handover, the paramedic will assist with the patient transfer as above.
6. *Checklist*. At 15 min after handover the paramedic will ask a member of the hospital team to confirm progress with key tasks: the status of the emergency brain scan request (if not yet performed) and stroke specialist review; confirmation of relevant medical history and medications; and ordering blood tests for clotting indices if relevant.
7. *Feedback*.After completion of the checklist, the paramedic will request feedback from a hospital clinician about the provisional pre-hospital diagnosis of stroke, the estimation of onset time and any other aspect of the assessment process.
8. *Completion*. After seeking the checklist and feedback information, the paramedic will complete and sign the study documentation. The paramedic will depart as per usual operational procedure. This will be within 15–30 min of hospital arrival and therefore compliant with NHS Commissioning Board guidance for ambulance to hospital handover of emergency admissions [24].

The paramedic will record a reason if study documentation is signed before completion of the PASTA pathway. Anticipated reasons are:

- The paramedic is no longer contributing towards the clinical care of the patient.
- A change in the clinical state of the patient making the PASTA pathway no longer appropriate (further details below).
- A stroke mimic condition is clearly identified during the initial hospital assessment, and the hospital team determine that it would no longer be appropriate to continue with a care pathway for suspected stroke (further details below).
- A specific request by the regional ambulance control centre that the ambulance crew should become available for another call due to the pressure on resources.

##### Permitted local variations to the PASTA pathway

Due to pre-existing variations in standard care clinical pathways and underlying healthcare service structures, there will be permitted variations in the different stages of the PASTA pathway as shown in Table 1.

**Table 1:** Permitted local variations to the PASTA pathway

PASTA component	Core content expected to be delivered	Local variation permitted
Information	Must use PASTA format	Any clinical information collection system (paper/electronic)
Pre-notification	Must be performed for all PASTA admissions	Paramedic call to ED Paramedic call to HASU Dispatch call to ED Dispatch call to HASU Pre-notification may or may not include patient-identifiable information
Handover	Must follow study format	Occurs in most suitable area for rapid handover and registration Receiving hospital team are ED and/or stroke service clinicians May include other relevant information for individual patients
Scan	Paramedic assists with transfer to scan if < 15 min since handover	Any appropriate hospital clinician accompanies patient Any brain imaging modality which would assist treatment decisions Transfer by ambulance or hospital trolley as long as no delay incurred
Assist	Patient transferred off ambulance trolley onto hospital trolley Paramedic offers assistance for initial tasks Ambulance crew prepares for departure	Team may not require assistance for each or all task(s) Ambulance operational conditions may shorten paramedic stay
Checklist	Paramedic enquires about progress from any hospital team member at 15 min since handover	Checklist completion should reflect usual local service procedures (e.g. telemedicine specialist review)
Feedback	Paramedic seeks feedback about provisional diagnosis and onset time	Any member of hospital team can provide feedback Paramedic can seek feedback on any aspect of the assessment The hospital team may not (yet) be able to provide feedback

##### Clinical deterioration

During the pre-hospital phase of the PASTA pathway, if the patient’s condition deteriorates, then the paramedic will re-evaluate whether it is still appropriate to continue with the pathway according to his/her professional judgement and standard clinical protocols. The reason for any deviation will be recorded, including the anticipated clinical scenarios of falling conscious level, seizure, hypotension and hypoglycaemia.

##### Stroke mimic conditions

Early identification of stroke patients is challenging, because other conditions (‘stroke mimics’) can create similar symptoms through different mechanisms, e.g. unwitnessed epileptic seizures and migraine. In clinical practice approximately 26% of patients who are suspected to have had a stroke in the pre-hospital setting receive a stroke mimic diagnosis following admission to hospital, brain imaging and stroke specialist review [25]. Thrombolysis treatment is inappropriate for these patients. If a stroke mimic condition becomes apparent during the initial hospital review, the hospital team will discontinue the IV thrombolysis assessment process as per standard clinical care.

#### Standard stroke care (comparison group)

Patients with suspected stroke symptoms attended by a control group paramedic receive standard stroke care as per current local ambulance and hospital clinical protocols, which are reinforced by national clinical guidelines and audit. The study does not provide comparison group paramedics with additional training or documentation to support information collection, clinical communication or processes after hospital arrival.

### Study participants

Patients are identified and approached about enrolment in the study after arrival at hospital (further details are provided in the subsequent section on ‘Participant identification, recruitment and consent’). Patients approached about enrolment meet the following criteria:

- Travelled to hospital with a study paramedic
- Aged 18 years and over
- Hospital specialist diagnosis of stroke
- Within 4 h of stroke onset (onset time determined by the hospital stroke team) when assessed by the study paramedic.

### Participant identification, recruitment and consent

Patients are identified and recruited and will consent to take part in this study after arrival at hospital and when the IV thrombolysis treatment assessment has been completed. There is no study enrolment process in the pre-hospital setting. The purpose of the study is to demonstrate that the PASTA pathway can expedite the clinical delivery of a treatment which is already known to be effective in reducing future disability but must be administered rapidly (IV thrombolysis). A formal research consent process performed by study paramedics or the admitting hospital team would delay hospital admission, brain imaging and/or IV thrombolysis treatment. Due to the time-dependent effect of IV thrombolysis, even a short delay could reduce the impact of the PASTA pathway. As the pathway does not involve a new treatment or technology but is attempting to expedite an existing hospital care process using a structured clinical assessment performed by paramedics, the risk of harm to intervention patients is low. Patients will still only receive IV thrombolysis treatment following review by a stroke specialist. Although they do not provide study information to patients, as per usual clinical practice, paramedics explain to patients about possible care processes which may occur in hospital.

In order to identify study-eligible patients, hospital research staff systematically review the ambulance and hospital records of all admitted patients with a confirmed hospital specialist diagnosis of stroke. Patients meeting the enrolment criteria are approached about taking part in the study.

Ideally, patients are approached during their inpatient stay such that a timely discussion about the study can be held. However, as some patients are discharged very early after admission and identification of eligibility may only occur after discharge, postal invitations to take part in the study are also used.

**Fig. 3:**
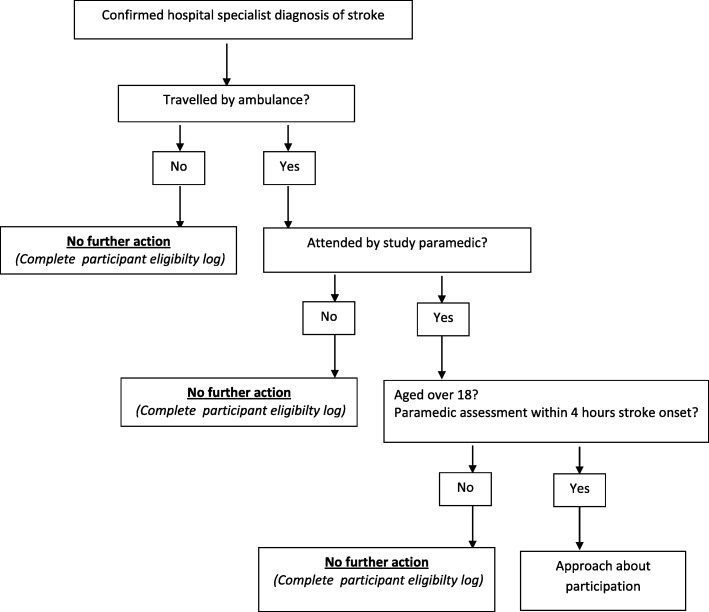
Identification of patients to approach for study participation

The participant eligibility assessment process is shown in Fig. 3.

Identification of confirmed stroke patients is facilitated by national clinical guidelines and audit, which mandate that all stroke patients should be admitted to the HASU within 4 h of hospital arrival, irrespective of the timing or the local service configuration [4, 8]. It is still possible that a small number of stroke patients attended by a study paramedic will not be admitted to the local HASU because of:

- Transfer to a neuroscience centre for possible surgical or neuro-interventional treatment
- Very quick recovery and discharge
- Bed availability in the local HASU
- Death before transfer from the ED to the HASU

In order to reduce the chance of these patients not being located in hospital, where possible, participating ambulance services send a regular site-specific report of all suspected stroke patients admitted by study paramedics to each participating site. Hospital research staff use this report to check the hospital diagnosis assigned. Where the diagnosis is confirmed stroke, other study eligibility criteria are reviewed and patients are approached about the study as appropriate.

Patients for whom a study paramedic suspects a stroke within 4 h of symptom onset (and as such should receive the PASTA pathway if seen by a trained intervention group paramedic), but who receive at hospital an alternative diagnosis (‘stroke mimics’) and/or an onset time which means the stroke commenced greater than 4 h ago are not approached about study enrolment. Although not contributing towards the study outcomes, the number and nature of stroke mimic conditions and whether or not IV thrombolysis was administered is recorded.

#### Consent

The consent process seeks permission for the use of routinely recorded healthcare data and for one study-specific assessment at day 90 after stroke (see the ‘Study data collection’ section later in the paper). In order to ensure that all eligible patients are provided with an opportunity to participate, several consent options are in use.

#### Consent for patients who can be approached about study participation during their inpatient stay

##### Consent for patients with mental capacity

For eligible patients with capacity to consent to research, hospital research staff approach the patient to discuss the study and provide a patient information sheet. After allowing sufficient time for a decision about whether to take part in the study and an opportunity to ask questions, consent is obtained in writing.

When a patient has mental capacity but is unable to sign the consent form (e.g. because of weakness of the dominant hand following stroke), consent is confirmed orally in the presence of a witness (an individual not otherwise involved in the trial), and the witness signs and dates the consent form on behalf of the participant.

##### Consent for patients with mild communication difficulties

For patients with mild communication difficulties due to the effects of stroke upon the use and understanding of language (aphasia), a set of ‘easy access’ study documentation is used. After allowing sufficient time for the information (including an ‘easy access’ patient information sheet) to be considered and an opportunity to ask questions, consent is obtained in writing using the ‘easy access’ consent form.

##### Consent for patients who lack mental capacity

It is anticipated that approximately one third of study-eligible patients will be unable to engage with an informed consent process due to the effects of stroke upon communication and cognition. As exclusion of this group would drastically reduce the clinical relevance of the study, if a patient has been identified as eligible but lacks the capacity to consent, a personal or professional consultee is approached.

Hospital research staff first attempt to identify an appropriate personal consultee (usually the next of kin) in order to discuss the study and provide a consultee information sheet. If a personal consultee is identified, after allowing sufficient time for him/her to consider the patient’s wishes and feelings and an opportunity to ask questions, the consultee is asked to complete a consultee declaration form if he/she believes the patient would have no objection to taking part in the study.

If an appropriate personal consultee cannot be located, an independent clinician (professional consultee) is asked to confirm that the patient lacks capacity for consent, and that study participation would not introduce a risk of harm or be against the patient’s wishes from what is known about the patient’s character and beliefs. The independent clinician signs an independent clinician declaration form concerning study participation.

As it is likely that the communication or cognitive difficulties that impeded a patient’s ability to provide consent will still be present at 90 days after stroke, where a personal consultee provided permission to enter the study, this person is contacted to complete the 90 day study-specific outcome questions on behalf of the patient. In cases where an independent clinician provided permission for study participation, only routinely available data are collected at 90 days. The participant is not contacted.

##### Consent and early mortality

The early mortality rate following acute stroke is approximately 10%. These patients are usually identified soon after admission and treated palliatively. However, unexpected deaths also occur. Exclusion of patients who die soon after admission would reduce the study’s relevance for the typical clinical stroke population.

When a patient has died, or if a formal palliative end of life care process has been started at the point when the patient is identified as eligible for the study, individual patient consent will not be possible, and it is likely to be distressing for a personal consultee to be approached regarding the research use of routinely collected healthcare data. The study-specific assessment at day 90 will not be relevant. Under these circumstances the local Principal Investigator (PI) signs an Early Mortality/Palliative Care Declaration Form to confirm that the patient has died or is in a formal palliative phase, and takes responsibility for the use of routinely collected healthcare data for this research project.

Acute stroke has many effects upon neurological function and consciousness which can fluctuate for several days or even weeks. Thus, there could be unusual instances where a patient who has been entered into the study using this Early Mortality/Palliative Care Declaration Form subsequently may show enough signs of improvement that supportive care is re-instated, and may still be alive at day 90. However, these are likely to be challenging clinical situations where seeking an alternative method of consent will be difficult because of the severe degree of remaining neurological impairment and the time elapsed since admission. In these unusual scenarios, an alternative method of consent will not be pursued, and the day 90 study-specific assessment will not be conducted. Routinely available data collected as part of standard clinical care will be retained as per the original PI declaration and used in the analysis.

##### Loss of capacity to consent to research during participation in the study

When participants have provided their own consent to take part in this research project, it is possible that they may temporarily (e.g. because of intercurrent illness) or permanently (e.g. because of further stroke) lose the capacity to assist with the 90 day study-specific assessment. On entering the study, participants are asked to nominate a personal consultee (a relative or close friend) who may be contacted to answer the 90 day study-specific questions on their behalf, should they be unable to undertake this assessment personally.

#### Consent for patients who are only identified as study eligible after discharge from hospital

Patients who are only identified as study eligible after hospital discharge receive an invitation letter, patient information sheet, consent form and pre-paid return envelope by post. Patients willing to take part in the study are asked to return a completed consent form. Invited patients who have not returned a consent form within 4 weeks receive one phone call from the local hospital research team. Thereafter, such patients will only be further contacted about the study if a consent form is returned.

**Fig. 4:**
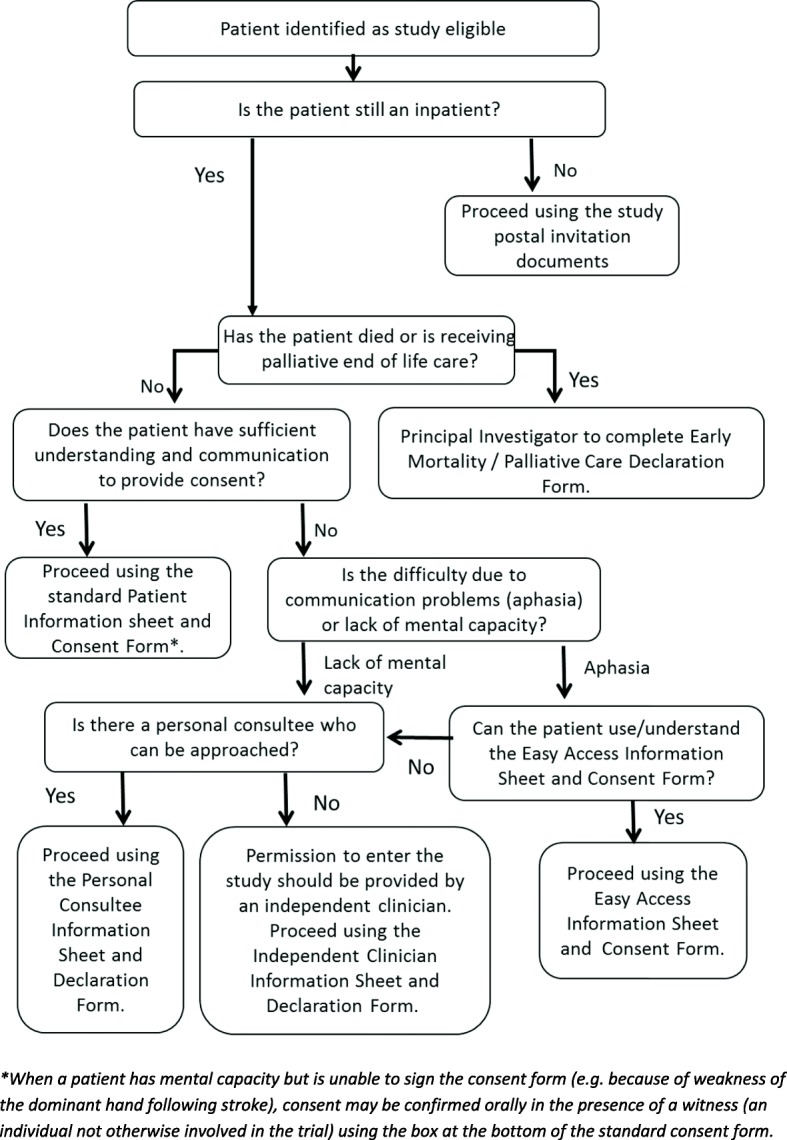
Decision process for study consent

Figure 4 summarises the decision process for obtaining study consent.

### Study data collection

Data are collected day 1 pre-admission, day 1 post-admission, day 2 (if IV thrombolysis is administered) and day 90 (+/− 7 days) post-stroke.

#### Day 1 pre-admission data

The following data are collected: suspected stroke diagnosis (FAST, paramedic judgement of stroke or query stroke); symptom onset or last known to be well time (paramedic judgement); pre-notification of receiving hospital; times of 999 call, ambulance on scene, ambulance left scene and hospital arrival; involvement of rapid response paramedic (i.e. arrived by car or motorbike ahead of the transporting ambulance): yes/no (intervention group only); time of transfer of care (also called handover) from the paramedic to the hospital team; time of paramedic signature of the completed ambulance clinical record; time paramedic is clear to respond to another incident.

The above data are predominantly transcribed from the routine ambulance clinical record onto a study-specific Case Record Form (CRF) by hospital research staff. However, some data items are not contained within the routine ambulance clinical record but held within computerised ambulance service dispatch systems. These data are obtained directly from the ambulance services. For participants travelling to hospital with an intervention paramedic trained to deliver the PASTA pathway, some of the preceding data are also recorded onto a study-specific paramedic CRF which additionally records delivery of the PASTA pathway.

#### Day 1 post-admission data

The following data are collected: demographic information (age, gender, pre-stroke mRS); date/time of hospital admission; first blood pressure reading on admission; first blood glucose reading on admission (capillary or serum glucose); stroke severity on admission (NIHSS); stroke onset or last known to be well time (stroke team judgement); current use of anticoagulant medication; previous medical history (stroke, TIA, heart failure, atrial fibrillation, diabetes, hypertension); hospital admission locations (ED, HASU, critical care, medical admissions ward, other ward); first brain imaging time, modality and result (CT/magnetic resonance imaging, infarction/primary intracerebral haemorrhage);thrombolysis treatment decision (yes/no, reason why IV thrombolysis not administered); date/time of IV thrombolysis (if received); use of other treatments (reversal of anticoagulation if haemorrhagic stroke, acute blood pressure lowering if haemorrhagic stroke or before IV thrombolysis, referral or transfer to neurosurgery, referral or transfer for intra-arterial treatment, use of intra-arterial treatments (yes/no, time of puncture)); entry into another clinical trial on day 1 after admission (yes/no, which one); date and time of admission to first HASU; date and time of assessment of swallow safety at first hospital.

#### Day 2 IV thrombolysis outcome (if administered)

The following data are collected: stroke severity (NIHSS) at 24–48 h after IV thrombolysis treatment (and/or intra-arterial treatment if received): standard post IV thrombolysis stroke severity measurement; complications (symptomatic intracranial haemorrhage/extracranial bleed/angio-oedema/other complication) within 48 h of IV thrombolysis treatment (and/or intra-arterial treatment if received): standard post IV thrombolysis complications measurement.

The days 1 and 2 post-admission data are all routinely collected clinical data. They are transcribed from routine medical records onto a study-specific hospital CRF by hospital research staff.

#### Day 90 (+/− 7 days) data

At day 90, data related to both hospital discharge from the continuous inpatient episode and health at 90 days after stroke are collected.

##### Data related to discharge due to completion of all inpatient care

The discharge data are as follows: died during this inpatient episode for stroke (yes/no); cause of death; date of death (if relevant); discharge date or record that the patient is still an inpatient at day 90 (yes/no); if still an inpatient at day 90, has there been stroke recurrence; discharge destination at completion of this inpatient episode (own home, different private address (e.g. relative) care home, other); if discharged to a care home, was the patient previously resident/not previously resident; rehabilitation arranged at discharge (none, early supported discharge team, community rehabilitation team, both); assistance with activities of daily living required at discharge (yes/no, if yes: what support received: informal carers/paid carers/both); if paid carers, then how many visits per week provided at discharge; dependency at discharge (mRS).

##### Data related to day 90 health

The day 90 health data are the following: died after stroke event discharge but prior to 90 days (yes/no); date of death (if relevant); cause of death (if available); readmission(s) to hospital since discharge: reason for admission(s), length of stay(s); readmission was due to stroke recurrence (yes, no); current (90 day) residence (hospital, home, different private address, care home, other); current (90 day) dependency (simplified questionnaire mRS [26]); community rehabilitation since discharge (therapy received (yes/no), received at home/hospital/both, number of weeks received, less than, equal to or greater than one appointment/week); current (90 day) assistance with activities of daily living (yes/no, if yes: what support received: informal carers/paid carers (how many visits per week)/both).

For all participants, confirmation that stroke remains the diagnosis assigned for the event where consent was obtained. If not, the new diagnosis assigned.

Day 90 data consist of both routinely collected clinical data and study-specific data. Routinely collected clinical data are transcribed from routine medical records onto a study-specific CRF. Study-specific data are collected by face-to-face interview, telephone interview or by postal questionnaire. Telephone interview with the participant or consultee is the main method and is undertaken by hospital research staff.

In instances where a participant/personal consultee cannot be contacted by telephone after one week of attempting to locate him/her, a postal questionnaire is used. A postal questionnaire is also used where telephone interview is not be possible (e.g. participant does not have a telephone). A single postal questionnaire with enclosed reply-paid envelope is mailed. It is also possible to collect 90 day study-specific data by face-to-face interview. This option is used at the discretion of research staff but predominantly when participants are currently hospital inpatients.

Where it has not been possible to obtain the 90 day study-specific data by 6 months after stroke, research staff use the routine hospital data administration systems to confirm whether the participant was alive or dead at 90 days. This is necessary because deaths in the community can take some time to appear in the routine hospital records. The mRS is scored 6 for death [27].

All study data are entered locally into a secure online database.

### Blinding

No specific blinding measures are in place. Paramedics will know the study group allocation due to the nature of the intervention. Participants will be informed about group allocation if they ask for this information. Research staff who conduct the 90 day research assessment may be unaware of treatment group allocation, but this will vary according to their involvement with participants on admission to hospital.

### Staff training and awareness

For paramedics based within stations randomised to deliver the PASTA pathway, study-specific training is provided via a securely hosted online video. This includes explanation of the study objectives, demonstration of the PASTA pathway processes, completion of trial documentation and responses to adverse events. After watching the video, paramedics complete an online multiple choice questionnaire knowledge assessment (MCQ). Paramedics who do not provide correct responses to all questions are invited to watch the online video again and repeat the MCQ. Paramedics who correctly answer all questions are identified as trained and issued study documentation. If paramedics based with stations allocated to delivering the intervention do not wish to assist with the study, they can opt out of training.

Paramedics based with stations randomised to continuing standard stroke care are informed that there is an ongoing study of pre-hospital assessment for stroke patients, but they are not given any further information about the study, and they are asked not to change their practice. Paramedics allocated to continuing standard stroke care can also opt out of the study if they wish.

To determine whether the characteristics of paramedics participating in each study group differ, the following will be obtained about individual randomised paramedics during the course of the study: age, gender, highest qualification, years qualified as a paramedic, years in service at employing ambulance service, NHS job band, job title. These characteristics will be obtained by means of an electronic survey.

Research staff based in hospitals receive study-specific training including identification, recruitment and consent of participants, data collection and the 90 day research assessment, as well as other study processes such as use of the online database.

Clinical staff at hospitals taking part in the study are made aware of the trial at service meetings and by email but do not receive additional protocol-related research training or documentation. Hospital services are not expected to change their standard local care process for patients undergoing IV thrombolysis treatment assessment as part of the study. They will continue to provide clinical care for all study patients consistent with the information and prompts provided by all paramedics within the context of the local service setting. If services change their configuration and standard care process during the study as a quality improvement initiative, this would apply to all stroke admissions and would not prevent the delivery of the paramedic-led PASTA intervention.

### Study withdrawal

No specific withdrawal criteria have been pre-set. Participants may withdraw from the study at any time for any reason. Data collected prior to withdrawal will be used in the study analysis unless the patient or the patient representative requests that this should not be the case. Should a decision to withdraw from the study be made, a reason for withdrawal will be sought, but participants can chose to withdraw without providing an explanation.

Occasionally, further clinical tests and information obtained result in a diagnosis of stroke being revised to an alternative diagnosis at a later date. Such patients will not be withdrawn from the study, as the initial care processes received were for a diagnosis of stroke.

### Safety evaluation

The PASTA intervention is designed to expedite standard stroke care and in particular IV thrombolysis treatment. Thrombolysis treatment is widely used in clinical practice, and its adverse events are well described. Administration cannot be repeated within 4.5 h of stroke onset, and all pharmacological action has been lost within 24 h. The PASTA intervention does not change the clinical criteria for IV thrombolysis administration; therefore, it is not possible for any adverse events after the emergency phase to be attributed to the study intervention.

However, to ensure that any potential adverse effects are recorded, this study is monitoring for serious adverse events (SAEs) for 7 days after study entry (paramedic assessment) and recording all deaths for the duration of a participant’s involvement in the study (until 90 days after stroke).

A study SAE form is completed for all events fulfilling the standard definition of an SAE with the expection of the following, which have been excluded from SAE reporting:

- Pre-planned hospitalisations and scheduled treatment for pre-existing conditions
- Events pre-defined as expected adverse events and monitored in the main dataset (death due to initial stroke, IV thrombolysis-related cerebral haemorrhage and stroke recurrence leading to new hospitalisation).

### Statistical analysis

Analysis is by ‘treatment allocated’; that is, primary and secondary effectiveness and safety outcome analyses will use the study group allocation of the station base for the attending paramedic.

The primary end point measure is the proportion of participants who received IV thrombolysis. The primary analysis will use logistic regression to compare the proportion of participants who received IV thrombolysis in the two arms of the study. Models will include clinically and statistically important covariates and factors, including (but not limited to) participant age and gender and admission time and date (used to define a covariate to reflect background trends).

Secondary outcomes, including stroke severity 24 h after IV thrombolysis (measured by an NIHSS score), inpatient mortality and dependency (mRS), will be analysed by appropriate ordinal and binary regression models, with appropriate adjustment for potential covariates and factors.

To address the second objective (that is, the impact of the intervention on the time intervals related to the emergency call), appropriate statistical models will be used. For time intervals between events which always happen (for instance, emergency call to hospital admission), linear models will be employed (after appropriate transformation), again adjusting for statistically significant covariates and factors. If the distributions of residuals are markedly non-normal, further transformation or bootstrapping will be considered. Residual diagnostics will also be used to identify outliers; identified outliers will be excluded and the analysis recalculated. For events which may or may not happen (for instance, emergency call to IV thrombolysis), outcomes will be analysed via binary regression models and survival analysis, comparing intervention and control arms using the framework of accelerated life testing; both approaches will again accommodate statistically significant covariates and factors.

Other clinical objectives will be addressed by appropriate descriptive statistical measures.

#### Sensitivity and subgroup analyses

Various sensitivity and subgroup analyses will be undertaken to supplement evidence from the primary analysis to help fully characterise the treatment effect. These will include (1) analysis by ‘treatment received’; (2) analysis of the study subgroup with a diagnosis of stroke unchanged at 90 days; (3) others which are considered important for understanding the study outcomes. Subgroup analyses will generally, but not exclusively, be undertaken for specific categories in factors with a significant relationship with the primary outcome measure. These analyses are either confirmatory or exploratory and, as the study is not statistically powered for multiple analyses, their results will be interpreted in this context.

#### Missing data

In general, we shall endeavour to adopt a consistent approach to missing data relating to both clinical and cost effectiveness, except where individual outcome measures require variation in that approach. For each variable, we shall summarise the frequency of missing data, which affects effective sample size and hence statistical power. If there is no reason to suspect that data are not missing completely at random (MCAR), we shall consider the use of appropriate imputation methods to ameliorate the problem of missing data; otherwise, the Trial Statistician and Chief Investigator will further discuss patterns in missing data.

#### Reporting

Outcome descriptions, summaries and comparisons will be expressed in accordance with appropriate Consolidated Standards of Reporting Trials (CONSORT) guidelines [28], including estimates with 95% confidence intervals to summarise two-tailed tests at the 5% significance level.

### Sample size

The sample size for this study postulates a change from 0.43 to 0.53 in the proportion of patients receiving IV thrombolysis (equivalent to a standardised statistical effect of 0.2). Using 90% power, 5% significance, an average cluster (patients per paramedic) of 5, an intra-cluster correlation coefficient of 0.02, an imbalance of 2 control patients per intervention patient and attrition of 1%, 1297 participants are required (865 control cases versus 432 intervention cases). An imbalance of control and intervention patients is included in this calculation because it was apparent early in the study that more control patients were being enrolled than intervention patients. This is because paramedics randomised to the intervention group are not considered part of the study unless they complete the study-specific training, and training rates are not as high as had initially been anticipated. This is resulting in more patients being conveyed to hospital by control than intervention paramedics. The emerging imbalance was considered during a sample size revision in 2017 (see the ‘Trial status’ section for further details about revisions to the study in 2017).

The final recruitment target is being kept under review, as should the attrition rate or imbalance ratio change during the ongoing course of the study, it may be necessary to adjust the target accordingly to achieve the required number of control and intervention analysable outcomes needed to maintain statistical power.

### Economic analysis

The primary economic analysis will be a within-trial evaluation to estimate the cost effectiveness of the enhanced role versus standard stroke care for increasing the proportion of patients receiving IV thrombolysis, and will report a cost per additional patient thrombolysed. In addition a cost utility analysis will be conducted to estimate cost per quality-adjusted life years (QALYs) gained.

#### Estimation of QALYs

Pre-stroke and 90 day mRS scores will be used to estimate QALYs. EuroQoL five dimension (EQ-5D) scores will be generated using mapping algorithms which have been developed by Whynes et al. [29] and Rivero-Arias et al. [30]. Mortality and utility values will be converted into QALYs using the area under the curve method, controlling for baseline health utility derived from mRS [31].

#### Estimating the resources used and costs for providing an enhanced paramedic role

The costs of paramedic time will be based on the equivalent hourly wage for the appropriate grades. The time taken to complete the training will be recorded by the computer-based training software. Training material productions costs will be excluded. The times from the ‘on scene’ to ‘handover’ and ‘clear to respond to another incident’ will be recorded and used to estimate additional ambulance service resource utilisation resulting from the enhanced paramedic role. The resource costs will be obtained from Ambulance Trusts and published sources including the Personal Social Services Research Unit (PSSRU) [32].

#### Estimating the consequences for resources resulting from an enhanced paramedic role

Data on the use of NHS inpatient resources will be collected for patients in both arms of the trial. These data will comprise brain imaging modality, provision of IV thrombolysis and length of stay in hospital. In addition, data about community rehabilitation therapy, social service involvement and non-elective episodes of secondary care post-discharge will also be collected at the days 1/2 and day 90 data collection points. Unit costs will be derived from routine sources for NHS and social care. The costs of providing the PASTA pathway for stroke mimic patients will be included but also presented separately. This will represent an economic worst-case scenario, because costs will be added, but it will be assumed that there is no benefit (or harm) to these individuals. If the intervention still appears cost effective in this scenario, then the trial conclusions would be strengthened.

#### Estimating cost effectiveness

Bootstrapping analysis of healthcare costs and outcomes will be used to estimate the mean differences and 95% confidence intervals between the enhanced role and standard care groups. The results will be presented as the point estimate of a mean incremental cost-effectiveness ratio (ICER) for the enhanced paramedic role versus standard care. The ICER will be calculated as a between-group difference in costs divided by the difference in effects (i.e. the proportions receiving IV thrombolysis and QALYs).

#### Missing resource use data

If there are data missing on post acute resource usage, essential items will be imputed from data present in the study for similar patients as defined by age, stroke severity and 90 day outcome [33]. Sensitivity analysis will quantify the degree of bias introduced by imputation.

#### Sensitivity analyses

Both stochastic and deterministic sensitivity analyses will be performed by non-parametric bootstrapping, including exploration of using different unit costs. Stochastic outputs will be presented as a cost-effectiveness plane and cost-effectiveness acceptability curves [34].

#### Long-term economic evaluation

In common with other economic evaluations, a further modelling analysis will be undertaken to extrapolate the results of the trial beyond the 90 days, and consider possible longer-term costs and effects. The model will be a microsimulation state transition model made up of three states (independent, dependent and death) to which costs and QALYs will be attached [35]. To estimate the longer-term effects of increased independence that may result from an enhanced paramedic role, the economic model will describe changes in dependency status over time. Outcomes after subsequent recurrent strokes will be estimated using a published decision analytic model (DAM) which predicts the outcome with and without IV thrombolysis [36]. The validity of the model will be confirmed against those 90 day outcomes available in the trial dataset.

#### Quantifying uncertainty in estimates of cost effectiveness

Both deterministic and probabilistic sensitivity analyses based on a Monte Carlo approach will be used to estimate the uncertainty in the economic model with results presented in the same format as the within-trial analysis. If after initial analysis it is found that the cost effectiveness of the intervention is sensitive to one or more inputs, an expected value of perfect parameter information (EVPPI) will be undertaken focussing on those inputs [37].

### Qualitative evaluation

A qualitative process evaluation is being undertaken to report upon patient experience and professional views (paramedics and hospital staff) regarding the acceptability and feasibility of the PASTA pathway in the clinical setting. Patients and professionals are invited to participate in semi-structured interviews:

1. *Medically stable patients who were attended by an intervention paramedic and provided their own consent to participate in the trial*.
  Following written informed consent, individual face-to-face interviews with a subgroup of patients enrolled in the trial are conducted by a qualitative researcher. Whenever possible, such interviews are arranged within 7 days of admission to facilitate recall of experiences and details.
2. *Paramedics*.
  Interviews are being conducted with intervention group paramedics, both those who have and have not completed the training. Interviewing non-trained intervention paramedics was introduced as a revision to the study in 2017 when it became apparent that training rates were not as high as anticipated. This made it important to understand barriers and facilitators to the deployment of the intervention should it be found to be of value for patient care.
  For intervention group paramedics who have completed training, purposive sampling aims to ensure representation of a range of times since use of the PASTA pathway (less or more than 30 days, no activations since training); paramedic seniorities (years of practice, employment title); base station setting (urban, rural); and experience of different patient admission routes (via ED, direct to HASU). For intervention paramedics who have not completed training, selection for interview aims to represent only each ambulance service and base station setting (urban, rural), as further information about non-trained paramedics is not captured during the study processes.
  According to availability, focus groups or individual interviews (in person or by telephone) are being conducted. Individual paramedics may be invited for interview on more than one occasion. For intervention group paramedics who have completed training, interviews focus on understanding views about delivery of the study intervention and the training process. For intervention group paramedics who have not completed training, interviews focus (as appropriate) on why training has not been undertaken and views on why they may or may not undertake training if the intervention was part of standard clinical care. In advance of the interview, paramedics receive an information sheet and consent form and are asked to return the consent form by post, email or in person. Due to the mobile nature of the paramedic workforce, it can be prohibitive to ask for return of completed forms prior to a telephone interview. If a telephone interview is planned and it is not practical to return the consent form by post or email in advance, verbal consent is taken and recorded before the interview commences. For face-to-face interviews or focus groups, a written consent form is completed.
3. *Hospital professionals*.
  Focus groups with hospital professionals working in the ED and stroke service who have witnessed the PASTA pathway are conducted. Hospital professionals are selected according to their clinical role (doctor, nurse, radiographer); local patient admission route (via ED or direct to HASU); and the local IV thrombolysis assessment approach (in person, telemedicine). According to availability, focus groups or individual interviews (in person or by telephone) are conducted. Individual professionals may be invited for interview on more than one occasion. In advance of the interview, hospital professionals receive a consent form and are asked to return it to the researcher conducting the interview by post, email or in person.

#### Data collection

Separate patient and professional interview topic guides are used to facilitate discussion. Data collection and analysis is occurring concurrently to allow for issues or themes identified in earlier interviews to be explored in more depth in subsequent interviews.

#### Data preparation and analysis

All interviews are digitally recorded and transcribed verbatim. In line with data protection legislation and research governance frameworks, all information pertaining to individuals/places is anonymised. The qualitative analysis will adopt a constructivist grounded theory approach [38]. Open, then focussed coding, will be undertaken, and emergent codes from the analysis of this stage will be presented to the wider research team. A suitable software package (e.g. NVivo) will be used to facilitate data analysis management.

### Confidentiality

Personal data are regarded as strictly confidential. Original paper CRFs containing study data are stored in the investigator site file at each research site. All study files are securely stored, and access is restricted to staff involved in the study. NHS research support staff at sites enter data from paper forms into a secure web-based electronic database. Data are entered using pseudo-anonymised participant identification codes and, except for the qualitative interview study where specific consent has been obtained for contact details to be given to the qualitative researcher, no identifiable information is transferred out of the local site. Access to the database is password protected and limited to staff at research sites or staff involved in managing the study overall.

The study complies with the Data Protection Act 1998, and Caldicott Guardian approval for use of data is sought in line with local requirements.

### Trial monitoring, quality control and quality assurance

The Chief Investigator has overall responsibility for the study conduct. The PIs are responsible for the day-to-day study conduct at their individual sites.

The trial is managed by a co-ordinating centre staff based at Newcastle University who provide day-to-day support for the sites and provide training through investigator meetings, site initiation visits and routine monitoring visits. A Trial Management Group (TMG) has been convened and meets regularly during the study.

Quality control is maintained through adherence to Newcastle Biomedicine Clinical Research Platform standard operating procedures (SOPs), the study protocol and research governance regulations. General monitoring of study conduct and data collected is performed by a combination of central review and site monitoring visits. The main areas of focus include consent, SAEs and essential documents in study files. All monitoring findings are reported and followed up with the appropriate persons in a timely manner.

The study may be subject to inspection and audit by Newcastle upon Tyne Hospitals NHS Foundation Trust under their remit as sponsor.

A Trial Steering Committee (TSC) has been convened to provide oversight of the trial. The TSC have agreed on a charter of operation and meet at least annually.

An independent Data Monitoring and Ethics Committee (DMEC) is established. Only the DMEC has access to unblinded outcome data before the trial ends. The DMEC have agreed on a charter of operation and meet at least annually.

### Dissemination of results

The study will be presented at national and international conferences and reported in peer-reviewed journals. Reports will be written for the study funder, sponsor and regulatory bodies. Anonymised data will be provided to research databases as requested (e.g. the Cochrane Collaboration) to enable future meta-analyses.

## Discussion

Improved timely access to acute stroke treatments is required. To date, most intervention has focussed on responses after patient admission to hospital, such as activation of a highly co-ordinated specialist team response. As the identification of and care for the majority of stroke patients is commenced pre-hospital by paramedics, simple enhancements to their routine processes may contribute towards improved access to specialist care, notably IV thrombolysis. This study will determine whether a Paramedic Acute Stroke Treatment Assessment (PASTA) care pathway improves access to IV thrombolysis and other aspects of stroke care.

## Trial status

The PASTA trial commenced recruitment in December 2015. The North East Ambulance Service, North West Ambulance Service and Welsh Ambulance Service are participating along with 12 NHS Hospital Trusts in England and 2 Health Boards in Wales. At the time of submission of this manuscript, 1038 patients were enrolled in the trial. Recruitment is scheduled to end in summer 2018, and results will be submitted for publication in 2019.

Protocol version 2 dated 31 October 2017 was used to prepare this manuscript. Amendments to the study which resulted in updating the protocol from version 1 to version 2 included the following:

- Changing the primary outcome (from a health outcome measured at day 90 by the mRS score to the proportion of patients receiving IV thrombolysis) and therefore modification of the sample size accordingly
- Addition of a group imbalance to the new sample size because of the incomplete uptake of study training by paramedics randomised to the intervention group
- Addition of a postal consent option
- Broadening the clinician interviews in the qualitative evaluation to include paramedics who had not completed the intervention training
- Alteration of the health economic analysis to align with the new primary outcome and inclusion of modelling of the health impact of the intervention

The change to the primary outcome decreased the required sample size. Decreasing the sample size was necessary, because the study recruitment rate was too low to achieve the original sample size in a reasonable timeframe.The new primary outcome is directly related to the previous primary outcome, as clinical trials have shown that receipt of IV thrombolysis carries a known probability of changing health outcomes. The new primary outcome is therefore reporting the direct impact of the intervention upon a patient care process which has predictable results for health outcomes.

## Additional file

Additional file 1: SPIRIT 2013 checklist: recommended items to address 1290 Q12 in a clinical trial protocol and related documents. (DOC 122 kb)

## Abbreviations

AE: Adverse event
AVPU: Alert Voice Pain Unresponsive
CRF: Case Record Form
CT: Computerised tomography
CTNT: Call to needle time
CTST: Call to scan time
CTU: Clinical Trial Unit
DMEC: Data Monitoring and Ethics Committee
DTNT: Door to needle time
DTST: Door to scan time
ED: Emergency Department
FAST: Face Arm Speech Test
IV rtPA: Intravenously administered recombinant tissue plasminogen activator
mRS: Modified Rankin Scale
NHS: National Health Service
NIHR: National Institute for Health Research
NIHSS: National Institutes of Health Stroke Scale
NRES: National Research Ethics Service
PI: Principal Investigator
PIS: Patient information sheet
QALY: Quality-adjusted life year
R&D: Research and Development
RCT: Randomised controlled trial
REC: Research Ethics Committee
SAE: Serious adverse event
SITS: Safe Implementation of Treatments in Stroke
TMG: Trial Management Group
TSC: Trial Steering Committee
